# Phase I and pharmacokinetic (PK) study of MAG-CPT (PNU 166148): a polymeric derivative of camptothecin (CPT)

**DOI:** 10.1038/sj.bjc.6601922

**Published:** 2004-06-08

**Authors:** D Bissett, J Cassidy, J S de Bono, F Muirhead, M Main, L Robson, D Fraier, M L Magnè, C Pellizzoni, M G Porro, R Spinelli, W Speed, C Twelves

**Affiliations:** 1 Department of Medical Oncology, University of Aberdeen, Aberdeen Royal Infirmary, Foresterhill, Aberdeen AB25 2ZN, UK; 2 Cancer Research UK Department of Medical Oncology, University of Glasgow, Beatson Oncology Centre, Western Infirmary, Glasgow G11 6NT, UK; 3 Cancer Research UK, Drug Development Office, London, UK; 4 Pharmacia and Upjohn, Nerviano, Italy

**Keywords:** MAG-CPT, polymeric, camptothecin, pharmacokinetics

## Abstract

Polymeric cytotoxic conjugates are being developed with the aim of preferential delivery of the anticancer agent to tumour. MAG-CPT comprises the topoisomerase I inhibitor camptothecin linked to a water-soluble polymeric backbone methacryloylglycynamide (average molecular weight 18 kDa, 10% CPT by weight). It was administered as a 30-min infusion once every 4 weeks to patients with advanced solid malignancies. The objectives of our study were to determine the maximum tolerated dose, dose-limiting toxicities, and the plasma and urine pharmacokinetics of MAG-CPT, and to document responses to this treatment. The starting dose was 30 mg m^−2^ (dose expressed as mg equivalent camptothecin). In total, 23 patients received 47 courses at six dose levels, with a maximum dose of 240 mg m^−2^. Dose-limiting toxicities were myelosuppression, neutropaenic sepsis, and diarrhoea. One patient died after cycle 1 MAG-CPT at the maximum dose. The maximum tolerated dose and dose recommended for further clinical study was 200 mg m^−2^. The half-lives of both MAG-CPT and released CPT were prolonged (>6 days) and measurable levels of MAG-CPT were retrieved from plasma and urine 4 weeks after treatment. However, subsequent pharmacodynamic studies of this agent have led to its withdrawal from clinical development.

Camptothecin (CPT) is the prototypic inhibitor of topoisomerase I. Although it was discovered in the 1970s, its clinical development was prevented by failure to devise a suitable formulation for this compound and the unpredictable and severe toxicity observed in early clinical trials, in particular myelosuppression, gastrointestinal toxicity, and haemorrhagic cystitis (Gottlieb *et al*, 1970; Muggia *et al*, 1972). In retrospect it is probable that these studies used mainly the open ring form of CPT, which is a relatively poor inhibitor of topoisomerase I but is more soluble than the active CPT lactone (Rivory and Robert, 1995). There is some conversion of the open form to CPT lactone in plasma and this explains much of the toxicity and the antitumour activity that was seen in these trials. The bladder toxicity is explained by conversion of the open ring to the closed ring form when CPT, which has been cleared by the kidneys, is exposed to low pH in the urine. It is all the more remarkable that despite these problems responses to CPT were observed in colorectal cancer, small bowel carcinoma, and melanoma in early clinical trials.

PNU-166148 (MAG-CPT) is a copolymer of *N*-(hydroxypropyl) methacrylamide, (20-*O*-(*N*-methacryloyl-glycyl-aminohexanoyl-glycyl)) camptothecin and *N*-(2-hydroxypropyl) methacryloyl glycinamide, which was developed by Pharmacia and Upjohn in an attempt to overcome problems in the clinical delivery of CPT. This polymeric derivative of CPT was designed specifically to increase cytotoxic drug delivery to the tumour. The macromolecule should preferentially accumulate within tumour tissue through extravasation via the abnormal tumour vasculature. The active but insoluble closed ring CPT molecule is covalently linked to the soluble MAG polymer through a glycyl-aminohexanoyl-glycyl spacer. The presence of this spacer modulates the intratumour release of CPT, requiring hydrolysis of the ester linkage which is pH and enzyme dependent, and may result in prolonged exposure of the tumour to low levels of CPT (Figure 1

**Figure 1:**
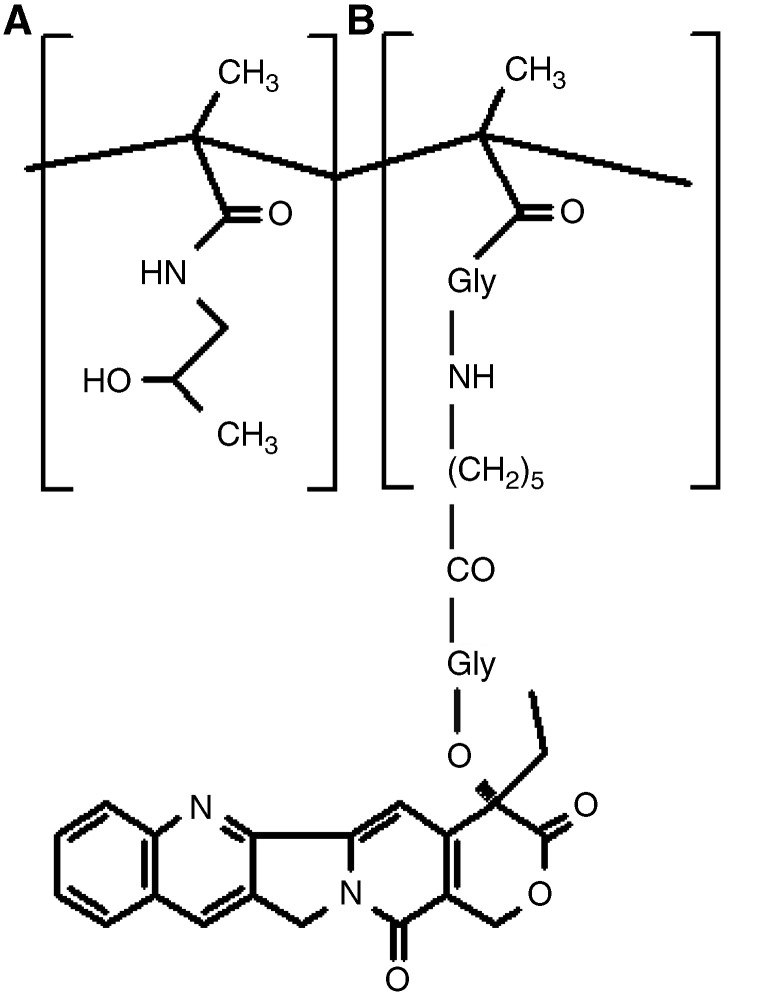
Structure of MAG-CPT. Free camptothecin is released by hydrolysis, which converts (**B**) to (**A**).

) (Conover *et al*, 1999).

Preclinical studies of MAG-CPT have shown that this compound is active in colon, stomach, pancreas, ovary, breast, lung, and melanoma xenograft models. The predicted delivery of the cytotoxic to tumour was confirmed in xenograft-bearing mice, with tumour concentrations of CPT exceeding plasma levels three days after a single i.v. treatment (Conover *et al*, 1998; Caiolfa *et al*, 2000). Toxicological studies in animals confirmed that the major toxicities of this compound were myelosuppression, diarrhoea, and transient impairment of renal and hepatic function.

The clinical development of MAG-CPT was driven by the combined promise of the established cytotoxic effect of CPT-mediated inhibition of topoisomerase I and the potential for enhanced delivery of CPT to tumour through a polymer delivery system. The principal objectives of this phase I study were (i) to determine the safety profile of MAG-CPT when administered as a single 30-min infusion every 4 weeks to adult patients with solid tumours; (ii) to determine the maximum tolerated dose (MTD) and recommend a safe starting dose on this schedule for phase II studies of MAG-CPT; (iii) to characterise the pharmacokinetic profile of MAG-CPT in terms of carrier-bound CPT and released-CPT; and (iv) to describe any antitumour activity observed in this phase I trial.

## PATIENTS AND METHODS

This was an open-label nonrandomised dose escalation study, conducted in sequential cohorts of patients from two institutions, Aberdeen Royal Infirmary, Aberdeen, and the Beatson Oncology Centre, Glasgow. It was performed under the auspices of the Cancer Research Campaign, London, with sponsorship by Pharmacia and Upjohn, Milan.

### Eligibility

All patients had histologically or cytologically proven advanced solid malignancy that was refractory to conventional therapy. Study entry criteria included age ⩾18 years, life expectancy of at least 12 weeks, adequate bone marrow reserves (absolute neutrophil count (ANC)⩾2000 *μ*l^−1^, platelets ⩾100 000 *μ*l^−1^), liver function tests (total bilirubin, AST and ALT) within normal limits unless known liver metastases when total bilirubin ⩽1.5 times upper limit of normal (NCI CTC grade 1), AST and ALT ⩽5 times upper limit of normal (NCI CTC grade 2), and adequate renal function (serum creatinine <1.5 mg dl^−1^, EDTA clearance >60ml min^−1^). The following exclusion criteria were applied: serious coexisting medical condition that could limit full compliance with the study, haematological malignancies, pregnant or breast feeding women, or fertile persons refusing to use contraceptives, known brain or leptomeningeal disease, previous treatment with irinotecan, more than two prior chemotherapeutic regimens for metastatic disease, high-dose chemotherapy requiring bone marrow transplantation or peripheral blood stem cell support, or radiotherapy to more than 25% of the bone marrow. All patients gave written informed consent according to local Ethics Committee guidelines.

### Drug administration and dosage

MAG-CPT has an average molecular weight of 18 kDa, polydispersity (*M*_w_/*M*_n_) 1.4, with <0.01 free CPT (wt%). The drug was supplied by Pharmacia and Upjohn as a sterile freeze dried powder for injection packaged in glass vials containing 0.5 g of MAG-CPT (equivalent to 50 mg of CPT). All doses of the investigational agent are expressed as mg of CPT equivalent. The drug was stored at room temperature. The vials were reconstituted with 10 ml normal saline and the dose to be administered was made up in 100 ml saline in a PVC infusion bag. A volumetric pump was used to deliver MAG-CPT as a 30-min i.v. infusion on day 1 of a 28-day cycle.

The starting dose for the study was 30 mg m^−2^, approximately one-tenth of the LD_10_ in mice. It was planned that dose escalation should proceed by 100% increments until the first occurrence of any NCI CTC Grade 2 drug-related toxicity (or Grade 1 neurotoxicity) was observed in the first treatment cycle. Thereafter, dose escalation was to continue according to a modified Fibonacci series. The dose escalation scheme could, however, be modified if considered appropriate based on acquired clinical and pharmacokinetic data. The MTD was defined as the highest dose at which at least three of six patients had dose-limiting toxicity (DLT). DLT was defined as (i) grade 2 neurotoxicity or grade 3 or 4 other nonhaematological toxicity, excluding grade 3 or 4 nausea or vomiting in patients who had not received a full antiemetic regimen (prophylactic antiemetics were not given in cycle 1), (ii) platelets <25 000 *μ*l^−1^, (iii) grade 4 neutropaenia (ANC <500 *μ*l^−1^) of at least 5 days duration or associated with fever (>38°C), or (iv) treatment delay exceeding 2 weeks caused by unresolved toxicity.

Patients were to be retreated at 28-day intervals for up to six cycles. A new course of treatment could begin if the ANC ⩾2000 *μ*l^−1^ and platelets ⩾100 000 *μ*l^−1^ and all other treatment-related toxicities had resolved to ⩽ grade 1 (grade 0 neurotoxicity). Treatment could be delayed for up to 2 weeks to allow resolution of toxicity. Escalation of MAG-CPT dose was not allowed for individual patients on the study. For patients with dose-limiting haematological toxicity in whom further treatment cycles were deemed appropriate these were to be given at the lower previous dose level.

Prophylactic treatment with antiemetics was not permitted during the first cycle of treatment unless nausea and vomiting that was clearly drug related had been observed at previous dose levels. Prophylactic growth factor support was not allowed. Patients requiring palliative radiotherapy were to be removed from the trial.

Patients were to be withdrawn from study if they completed six treatment cycles, developed progressive disease, failed to recover from toxicity despite a 2-week delay in therapy, if changes in their medical status made them no longer eligible for the study, or at the discretion of the investigator, or at the request of the patient.

### Pretreatment and on-treatment assessments

Prior to treatment all patients had a complete medical history and physical examination, FBC, serum chemistry, urinalysis, EDTA clearance, 12 lead ECG, chest X-ray, and tumour assessment by physical examination, CT scan, or ultrasonography as appropriate. During the course of the study patients were reviewed in the clinic every week with toxicity assessment, physical examination, FBC, and serum chemistry. Tumour assessment by imaging was repeated every three cycles. Chest X-ray and ECG were repeated at the end of study.

### Pharmacokinetics

Blood samples were taken from all patients following their first course of MAG-CPT predose, halfway through infusion, at the end of infusion, and at 10 min, 30 min, 1 h, 2 h, 4 h, 8 h, between 10 and 16 , 24, and 48 h after the end of infusion. Further blood samples were taken at weekly intervals while the patients remained on study. For each sample two aliquots of plasma were separated, stored at −20°C, and shipped on dry ice to Pharmacia and Upjohn, Milan, for the analysis.

A high-performance liquid chromatographic (HPLC) method with fluorescence detection was used for the determination of CPT (intact lactone and carboxylate) concentrations as CPT released *in vivo* from MAG-CPT (denoted as released-CPT) and, indirectly, as CPT bound to the carrier (denoted carrier-bound CPT) (Schoemaker *et al*, 2001). The carrier-bound CPT concentrations (expressed as ng eq CPT/ml) were obtained by subtracting the released CPT levels from the total levels of CPT obtained after alkaline hydrolysis of MAG-CPT. To avoid release of CPT during sample manipulation, the aliquot of plasma used for the determination of released CPT was immediately acidified. Plasma pharmacokinetic parameters of both carrier-bound and released-CPT were calculated using noncompartmental methods with the aid of WinNonlin (version 2.1, Scientific Consulting Inc., 1996). Descriptive statistics (mean, s.d.) were calculated for plasma levels at each time point and for pharmacokinetic parameters calculated after each dose level.

Urine samples were collected from all patients on the day of drug administration following the first course of MAG-CPT and weekly thereafter. Urine samples, stored at −20°C, were shipped on dry ice to Pharmacia and Upjohn, Milan, for the determination of total CPT by HPLC.

## RESULTS

A total of 23 patients were registered between January 1999 and April 2000, and their characteristics are outlined in Table 1

**Table 1:** Patients characteristics

**Characteristic**	**Number**
No. of patients	23
*No. of assessable courses*	47
Median	2
Range	1–6
	
*Sex*	
Male	15
Female	8
	
*Age*	
Median	62
Range	33–75
	
*ECOG performance status*	
0	5
1	18
2	0
	
*Prior therapy*	
Chemotherapy	21
Radiotherapy	13
Hormone therapy	2
	
*Tumour type*	
Colorectal	6
Unknown primary	4
Head and neck	2
NSCLC	2
Renal	2
Adrenal	1
Cervical	1
Oesophageal	1
Ewing's	1
Gastric	1
Mesothelioma	1
Prostatic	1

. They received 47 courses of MAG-CPT through six dose levels ranging from 30 to 240 mg m^−2^, and all courses were fully assessable for toxicity. The number of cycles delivered at each dose level is summarised in Table 2

**Table 2:** Dose levels and cycles of MAG-CPT delivered

**Dose level (mg m^−2^)**	**% Dose increment**	**No. of patients**	**No. of cycles**	**Dose reductions**
30		3	2,1,2	
60	100	3	1,3,1	
120	100	3	2,3,2	
240	100	3	1,1,1	Two patients re-treated at 120 mg m^−2^
170	−30	3	2,1,2	
200	18	8	2,2,6,1,2,1,2,1	

. The median number of cycle administered per patient was 2 (range 1–6). All treatment cycles were administered on time at the planned dose with the exception of cycle 2 in two patients treated at 240 mg m^−2^ when a 50% dose reduction was applied because of DLT in cycle 1.

No significant drug-related toxicities were noted at the 30, 60, or 120 mg m^−2^ dose levels, allowing dose escalations of 100%. Dose-limiting haematological and nonhaematological toxicities were observed in all three patients treated at 240 mg m^−2^. Subsequent cohorts of patients were treated at intermediate dose levels of 170 and 200 mg m^−2^ in order to define a safe dose for phase 2 studies.

### Toxicities

Nausea and vomiting grade 1 was observed sporadically through dose levels 30–120 mg m^−2^. No other significant treatment-related toxicities were observed at these dose levels.

The first patient treated at 240 mg m^−2^ had extensive metastatic colorectal carcinoma with lung and liver metastases. His prior therapy included adjuvant 5FU and folinic acid chemotherapy, pelvic radiotherapy, further chemotherapy with 5FU and folinic acid for metastatic disease, and palliative radiotherapy for rib metastases. Following cycle 1 MAG-CPT he developed grade 4 myelosuppression and grade 2 diarrhoea. He was admitted on day 12 with neutropaenic sepsis (WCC 0.07 *μ*l^−1^) and thrombocytopaenia (platelets 1 *μ*l^−1^). He deteriorated rapidly despite supportive care with i.v. antibiotics, platelets, and blood transfusion (Hb 9.0 g dl^−1^ on admission). He died of multiorgan failure less than 24 h after admission.

The second patient treated at this dose level had malignant mesothelioma. Following cycle 1 she developed grade 3 diarrhoea and grade 3 myelosuppression. She was also admitted with neutropaenic sepsis (nadir neutrophils 0.85 *μ*l^−1^, platelets 88 *μ*l^−1^ on day 10), but rapidly recovered from this with supportive care and i.v. antibiotics. Haematological toxicity had completely resolved by day 22. She received a second cycle of MAG-CPT at a reduced dose (120 mg m^−2^) with no drug-related toxicity of note. The third patients treated at 240 mg m^−2^ had metastatic squamous carcinoma of unknown primary. Following cycle 1 she had grade 3 diarrhoea with no myelosuppression. In view of the toxicity experienced by the previous two patients, she was also retreated at the lower dose of 120 mg m^−2^ and had no further drug-related toxicity of note. The MTD for MAG CPT in this study was therefore 240 mg m^−2^.

No significant drug-related toxicity was observed in the three patients treated at 170 mg m^−2^. Only one of the eight patients treated at 200 mg m^−2^ had grade 2 dysuria and haematuria during cycle 2. No significant haematological toxicity occurred at either of these intermediate dose levels. Accordingly, 200 mg m^−2^ was the dose recommended for phase II studies.

### Antitumour activity

No objective antitumour responses were seen. At 60 mg m^−2^, regression of cutaneous metastases was observed in a patient with renal carcinoma. However, this patient had progressive disease with the development of brain metastases.

### Pharmacokinetics

The main pharmacokinetic parameters for both carrier-bound and released-CPT at all dose levels are summarised in Tables 3

**Table 3:** Pharmacokinetic parameters of carrier-bound CPT in plasma following cycle 1 MAG-CPT

**Dose (mg m^−2^)**	***C*_end inf_ (*μ*g ml^−1^)**	***t*_1/2*Z*_ (h)**	**AUC_0–_ (*μ*g h ml^−1^)**	**CL (ml h^−1^ m^−2^)**	***V*_ss_ (ml m^−2^)**
30	15.5 (3.8)	216 (22)	976.2 (163.7)	31.4 (5.8)	7841 (1903)
60	30.4 (1.6)	132 (49)	1760.7 (533.6)	36.2 (10.6)	5297 (529)
120	64.0 (8.4)	219 (16)	5747.6 (596.9)	21.0 (2.1)	5710 (722)
170	78.6 (3.6)	188 (29)	6559.1 (497.9)	26.0 (1.9)	6289 (524)
200	111.9 (35.2)	237 (37)	9305.3 (2170.5)	22.3 (4.7)	6481 (1883)
240	126.7 (21.5)	155 (*n*=2)	10 667.5 (*n*=2)	23.0 (*n*=2)	4326 (*n*=2)

Values are mean (standard deviation).

*C*_end inf_=concentration at the end of infusion; AUC_0−∞_=area under the plasma concentration *vs* time curve extrapolated to infinite time; CL=systemic clearance; *V*_ss_=volume of distribution at steady state; *t*_1/2*z*_=apparent terminal half-life.

and 4

**Table 4:** Pharmacokinetic parameters of released-CPT in plasma following cycle 1 MAG-CPT

**Dose (mg m^−2^)**	***C*_max_ (*μ*g ml^−1^)**	***T*_max_ (h)**	***t*_1/2*Z*_ (h)**	**AUC_0–∞_ (*μ*g h ml^−1^)**
30	0.060 (0.039)	25 (0)	168 (107)	10.0 (4.5)
60	0.141 (0.087)	25 (0)	185 (97)	24.3 (12.0)
120	0.196 (0.079)	15 (9)	216 (58)	37.8 (20.0)
170	0.127 (0.023)	25 (0)	224 (23)	31.5 (10.8)
200	0.230 (0.133)	25 (11)	209 (64)	43.8 (25.5)
240	0.589 (0.105)	28 (19)	228 (*n*=2)	73.3 (*n*=2)

Values are mean (standard deviation).

*C*_max_=maximum concentration; *T*_max_=time to maximum concentration; AUC_0–∞_=area under the plasma concentration *vs* time curve extrapolated to infinite time; *t*_1/2*z*_=apparent terminal half-life.

. Figure 2

**Figure 2:**
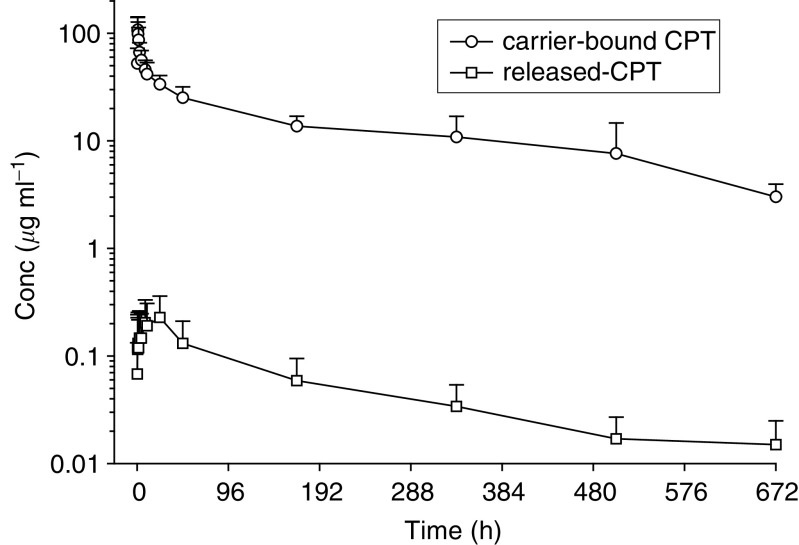
Mean (s.d.) plasma concentration–time curves of carrier-bound CPT and released-CPT following 200 mg m^−2^ dose.

shows mean (s.d.) plasma concentration time curves of both compounds for patients treated at 200 mg m^−2^. Carrier-bound CPT was characterised by low clearance and a volume of distribution of the same order as the volume of biological fluids. Plasma pharmacokinetic parameters of carrier-bound CPT appeared dose independent over the range of tested dose levels.

**Figure 3:**
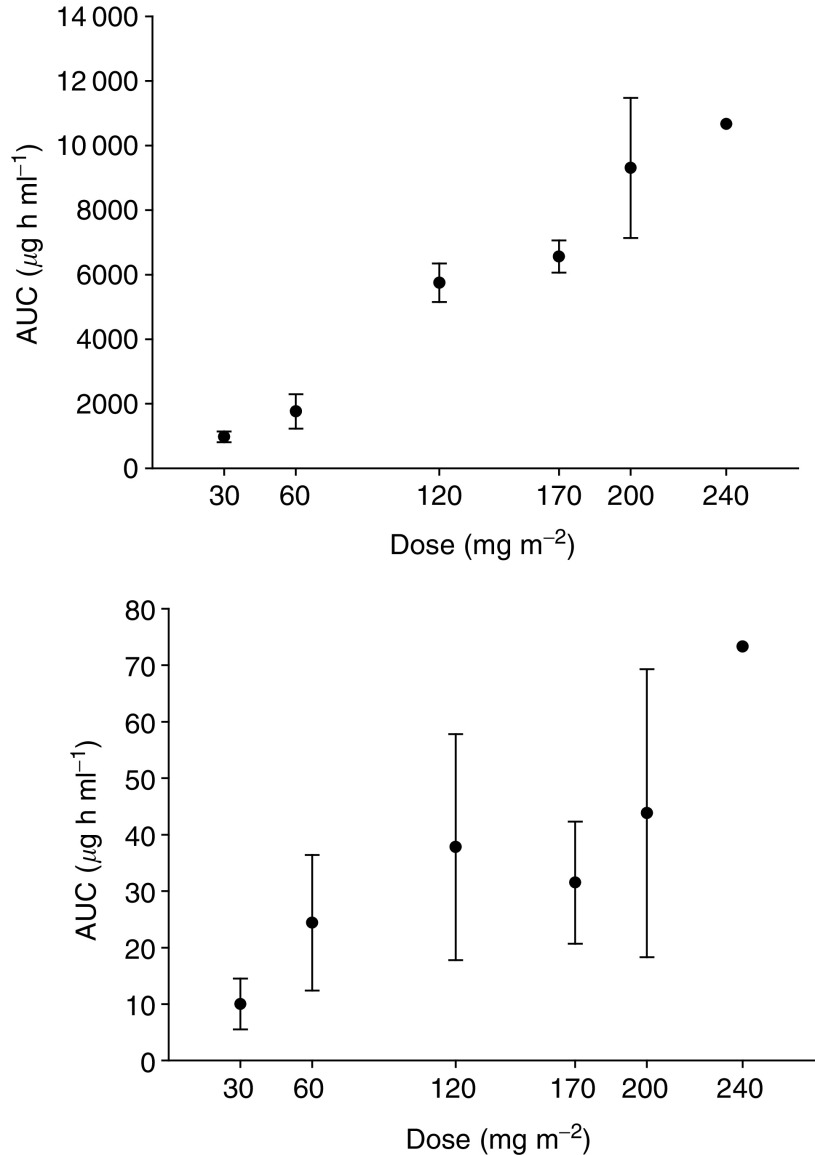
Mean (s.d.) AUC_0−∞_ of carrier-bound CPT (upper panel) and released-CPT (lower panel) as a function of dose.

The *C*_max_ for released CPT was achieved about 24 h after dosing at each dose level. After the peak, released-CPT levels decayed with a terminal half-life similar to that of carrier-bound drug (range 97–291 h). Plasma levels of released-CPT accounted for 0.01–4.9% of carrier-bound drug concentrations (median 0.28%). Carrier-bound CPT and released-CPT AUC_0−∞_ as a function of dose are reported in Figure 3

. Overall, no substantial deviation from dose proportionality was apparent. Both carrier-bound and released-CPT were detectable in plasma up to 4 weeks after treatment. However, in patients who received multiple cycles of MAG-CPT there was no evidence of drug accumulation. Following cycle 2 MAG-CPT at 200 mg m^−2^, the mean plasma level of released-CPT decreased from 0.113 *μ*g ml^−1^ on day 8 to 0.025 *μ*g ml^−1^ on day 29.

Table 5

**Table 5:** Urinary parameters of total CPT following cycle 1 MAG-CPT

**Time interval**	**0–8 h**		**8–24 h**		**0–24 h**	
**Dose (mg m^−2^)**	**Ae (mg)**	**fe (% dose)**	**Ae (mg)**	**fe (% dose)**	**Ae (mg)**	**fe (% dose)**
30	31.1 (5.2)	58.4 (5.6)	10.2 (1.8)	19.1 (1.7)	41.3 (7.0)	77.5 (7.3)
60	42.6 (13.2)	37.7 (10.3)	18.9 (4.6)	17.5 (7.6)	61.6 (9.2)	55.2 (11.8)
120	133.0 (52.4)	59.4 (23.6)	36.5 (21.5)	16.5 (10.0)	169.5 (64.1)	76.0 (29.5)
170	125.1 (49.2)	39.0 (16.2)	46.1 (18.5)	14.0 (5.2)	171.2 (47.3)	53.0 (15.3)
200	322.8 (178.7)	30.5 (7.5)	97.8 (51.2)	25.7 (15.0)	193.6 (29.4)	49.3 (5.3)
240	777.8 (659.6)	45.3 (26.1)	104.6 (92.1)	22.4 (18.7)	309.3 (59.3)	67.7 (9.5)

Values are mean (standard deviation).

Ae=amount of total CPT excreted in urine. For each time interval this was calculated as the product of the measured urinary concentration and the urinary volume; fe=amount of total CPT excreted in urine expressed as percentage of dose administered.

summarises the data on the amount of total CPT excreted in the urine within 24 h of treatment with MAG-CPT. At all doses tested, on average, at least 50% of the administered dose of total CPT was recovered in the urine during the first 24 h. Drug could be detected at all dose levels in the urine of most patients 28 days postdosing. In patients treated at 200 mg m^−2^ the mean urinary concentration of total CPT decreased from 2.8 *μ*g ml^−1^ in week 2 to 0.5 *μ*g ml^−1^ in week 4 of the first treatment cycle.

## DISCUSSION

This study has shown that it is possible to deliver higher doses of camptothecin as a polymeric derivative compared with the parent compound. Moreover, at the recommended dose of 200 mg m^−2^, the toxicities of MAG-CPT were predictable and manageable. The dose-limiting side effects that were observed in this study, mainly myelosuppression and diarrhoea, were those predicted from preclinical toxicology. However, the highest dose of 240 mg m^−2^ is clearly above the tolerable dose of MAG-CPT with all three patients experiencing life-threatening toxicities. From the experience outlined in this study we would recommend that the appropriate dose for Phase II studies of MAG-CPT is 200 mg m^−2^ once every 4 weeks.

The design of this compound was intended to result in preferential and prolonged exposure of the tumour to camptothecin with relatively low levels of normal tissue drug exposure. MAG-CPT does result in some interesting changes in the pharmacokinetics of camptothecin with a prolonged half-life for both carrier-bound and released-CPT. It is however not clear from this study whether this translates into a pharmacodynamic benefit, and certainly no sign of significant antitumour activity was observed in this study. The toxicities observed within this study, namely myelosuppression, neutropaenic sepsis, and diarrhoea, were similar to those observed with the parent compound camptothecin.

This is the second clinical study of MAG-CPT to be reported. The earlier study examined an alternative schedule of delivery, with treatment on the first 3 days of a 28-day cycle (Schoemaker *et al*, 2002). Neither myelosuppression nor diarrhoea was dose limiting in this study. Instead bladder toxicity, with dysuria and haematuria, was dose limiting at 130 mg m^−2^ day (390 mg m^−2^ per 4 week cycle). There was no evidence of antitumour activity of MAG-CPT in this trial.

Several macromolecular systems have been developed with a cytotoxic drug complexed or covalently bound to the carrier. In preclinical studies this approach clearly alters the pharmacokinetics of the cytotoxic but also enhances drug delivery to the tumour, a phenomenon termed the Enhanced Permeability and Retention (EPR) effect (Matsumura and Maeda, 1986; Seymour, 1992). It is thought that EPR is due to the discontinuous nature of the tumour endothelium allowing extravasation of the macromolecule and also the lack of effective lymphatic drainage within the tumour. The first such cytotoxic-copolymer conjugate to undergo clinical evaluation was PK1. This comprises doxorubicin bound to the water-soluble *N-*(2-hydroxypropyl) methacrylamide copolymer by a peptidyl linker. This markedly altered the pharmacokinetics of doxorubicin and allowed several fold higher doses of doxorubicin to be administered compared with the parent compound (Vasey *et al*, 1999). In particular, there was no evidence of cardiotoxicity and no polymer-related toxicities. The EPR effect was demonstrated for this compound in the clinical trial, when radiolabelled drug was demonstrated to accumulate within tumour. Moreover, PK1 showed evidence of activity in refractory cancers in the phase I trial.

MAG-CPT differs from PK1 with regard to its polymer and spacer as well as the cytotoxic moiety, but the aim of its design was similar, namely to achieve preferential delivery of the cytotoxic into tumour tissue. The observed toxicities in the two clinical trials of MAG-CPT may be explained by hydrolysis of the ester linkage between CPT and polymer either in blood or during urinary excretion, exposing normal tissues to free CPT. In contrast, the peptidyl linker of PK1 was designed for intratumoural cleavage by lysosomal cysteine proteases and appears successful in this role (Duncan *et al*, 2001).

At the time of completion of the phase I studies of MAG-CPT, a ‘proof of principle’ trial was initiated by Pharmacia in order to measure the delivery of camptothecin to tumour by MAG-CPT. A total of 10 patients with localised colorectal cancer received a single dose of MAG-CPT 60 mg m^−2^ up to 7 days prior to elective surgery for resection of their primary tumour (Sarapa *et al*, 2003). Carrier-bound and released-CPT were measured in plasma, tumour, and adjacent normal bowel. Although MAG-CPT was delivered in measurable concentration to the tumour, disappointingly the concentrations of released CPT were consistently lower in tumour than in normal colon. In addition, the concentration of CPT in blood exceeded that within the tumour even at 7 days after injection, and there was no evidence of preferential retention of the cytotoxic within the tumour. Based on the results of the phase I studies and the intratumoural kinetic study, Pharmacia made a strategic decision to discontinue further clinical development of MAG-CPT. However, development of other therapeutic molecules with polymeric delivery systems continues and it seems likely that the clinical promise of this approach will be fulfilled in the future.
